# Measuring Caloric Intake at the Population Level (NOTION): Protocol for an Experimental Study

**DOI:** 10.2196/12116

**Published:** 2019-03-12

**Authors:** Elisa Fuscà, Anna Bolzon, Alessia Buratin, Mariangela Ruffolo, Paola Berchialla, Dario Gregori, Egle Perissinotto, Ileana Baldi, NOTION Group

**Affiliations:** 1 Department of Cardiac, Thoracic and Vascular Sciences University of Padova Padova Italy; 2 Department of Clinical and Biological Sciences University of Torino Torino Italy

**Keywords:** wearable device, dietary monitoring, calorimetric assessment, machine learning, big data

## Abstract

**Background:**

The monitoring of caloric intake is an important challenge for the maintenance of individual and public health. The instruments used so far for dietary monitoring (eg, food frequency questionnaires, food diaries, and telephone interviews) are inexpensive and easy to implement but show important inaccuracies. Alternative methods based on wearable devices and wrist accelerometers have been proposed, yet they have limited accuracy in predicting caloric intake because analytics are usually not well suited to manage the massive sets of data generated from these types of devices.

**Objective:**

This study aims to develop an algorithm using recent advances in machine learning methodology, which provides a precise and stable estimate of caloric intake.

**Methods:**

The study will capture four individual eating activities outside the home over 2 months. Twenty healthy Italian adults will be recruited from the University of Padova in Padova, Italy, with email, flyers, and website announcements. The eligibility requirements include age 18 to 66 years and no eating disorder history. Each participant will be randomized to one of two menus to be eaten on weekdays in a predefined cafeteria in Padova (northeastern Italy). Flows of raw data will be accessed and downloaded from the wearable devices given to study participants and associated with anthropometric and demographic characteristics of the user (with their written permission). These massive data flows will provide a detailed picture of real-life conditions and will be analyzed through an up-to-date machine learning approach with the aim to accurately predict the caloric contribution of individual eating activities. Gold standard evaluation of the energy content of eaten foods will be obtained using calorimetric assessments made at the Laboratory of Dietetics and Nutraceutical Research of the University of Padova.

**Results:**

The study will last 14 months from July 2017 with a final report by November 2018. Data collection will occur from October to December 2017. From this study, we expect to obtain a series of relevant data that, opportunely filtered, could allow the construction of a prototype algorithm able to estimate caloric intake through the recognition of food type and the number of bites. The algorithm should work in real time, be embedded in a wearable device, and able to match bite-related movements and the corresponding caloric intake with high accuracy.

**Conclusions:**

Building an automatic calculation method for caloric intake, independent on the black-box processing of the wearable devices marketed so far, has great potential both for clinical nutrition (eg, for assessing cardiovascular compliance or for the prevention of coronary heart disease through proper dietary control) and public health nutrition as a low-cost monitoring tool for eating habits of different segments of the population.

**International Registered Report Identifier (IRRID):**

DERR1-10.2196/12116

## Introduction

Obesity affects 650 million people worldwide and is strongly related to cardiovascular diseases, which are the main cause of death in Western countries [1,2]. Therefore, the monitoring of caloric intake and energy expenditure are crucial challenges for the maintenance of individual and public health. Monitoring of diet requires daily recording of each food consumed and its energy content (and sometimes other macronutrients). The instruments used for this purpose include food questionnaires, food diaries, and telephone interviews [3-5]. Questionnaires are self-administered instruments that are low cost and easy to use. Despite some recent attempts to increase the immediacy of their use, these tools keep showing limited accuracy [6]. The reason is that the quality of data collected depends on the precision of the respondent in reporting their food intake and relative serving size [1,2,7]. In addition, long-term diet monitoring by self-evaluation increases the risk of underestimating energy intake and reporting incorrect or incomplete information [8].

With advances in technologies, studies have started to employ electronic wearable devices for monitoring caloric intake [9]. Wearable devices seem to be the future of research on nutrition since they are expected to reduce the error related to subjective evaluation, guaranteeing an adequate, objective, and reliable estimate of caloric intake [4,9].

Wearable devices equipped with motion sensors usually detect feeding-related gestures, translate gestures into a number of bites, and then bites into energy intake through the use of algorithms whose rationale is based on the existence of a relationship between the number of bites and the number of calories eaten [9]. For example, the Bite Counter is worn on the wrist and counts the number of bites taken by the movement of the arm or wrist [10]. It essentially consists of a watch that records the movements associated with the action of biting, counting them. In accordance with recent studies, the bite counters currently in use seem to be accurate in identifying bites, but not in associating bites to the corresponding caloric intake [11,12].

The main reason they are poor in associating bites to calories is because of their poor analytics, which are usually not well suited to manage the massive sets of data generated from these devices and with a limited accuracy in predicting caloric intake [8,13-16]. Technology alone is not effective unless smart data analytics complements it. Using the cutting-edge advances in machine learning and through the dynamic exploitation of big raw data coming from the real-life use of wearable sensors, this project aims to provide a ready-to-implement algorithm for monitoring caloric intake accurately. These massive data flows will provide a detailed picture of real-life conditions and will be analyzed through an up-to-date machine learning approach with the aim to accurately predict the caloric contribution of individual eating activities independent of the wearable device chosen. This protocol describes the experimental study set up for the development of an algorithm able to estimate the caloric intake from kinetic data.

## Methods

This section describes the protocol of the study “Measuring Caloric Intake at the Population Level” (acronym NOTION), approved by the Bioethics Committee of the University of Torino, Torino, Italy, on July 12, 2017 (#256091).

### Data Collection

The study will last 14 months from July 2017 with a final report by November 2018. Data collection will occur from October to December 2017.

#### Volunteer Recruitment

The study will capture four individual eating activities outside the home over a 2-month period. Twenty healthy Italian people will be recruited from the University of Padova with email, flyers, and website announcements. The eligibility requirements include age 18 to 66 years, no eating disorder history, and absence of allergies and food intolerances. Potential participants will complete a Web-based initial form to collect a preliminary assessment of the fulfillment of the inclusion criteria. Those who appear eligible will meet with the research team members who will describe the study, answer questions, and obtain written informed consent to determine final eligibility. Participants will receive an eating activity schedule, a device, and written instructions.

Each participant will be required to eat a minimum of four meals on weekdays in a predefined cafeteria in Padova (northeastern Italy).

#### Collection of Anthropometric and Nutritional Information

To collect information about dietary habits and physical activity, a standard questionnaire will be administered to all enrolled participants [17] also considering the available online tools [18]. Furthermore, to have a baseline view on body composition of the participants, some anthropometric traits will be recorded. Height (m) and weight (kg) will be measured using a SECA 220 weighting scale with stadiometer (maximum capacity 220 kg; accuracy: 0.1 kg; 0.001 m), and the body mass index will be derived (kg/m^2^); body density (g/cc) will be calculated by Durnin and Womersley equations, adjusted by age, using the logarithmic sum of four skinfolds using a Holtain 610 Caliper (accuracy: 0.1 mm): biceps, triceps, subscapularis, and iliac [8]. The Siri equation will be then used to calculate fat mass (%) and fat-free mass (%) [8]. The waist-hip ratio will be calculated by measuring their circumferences (in cm). Skinfold measurements will be performed by the dieticians’ staff as recommended by official guidelines [9].

### Experimental Phase

**Figure 1:**
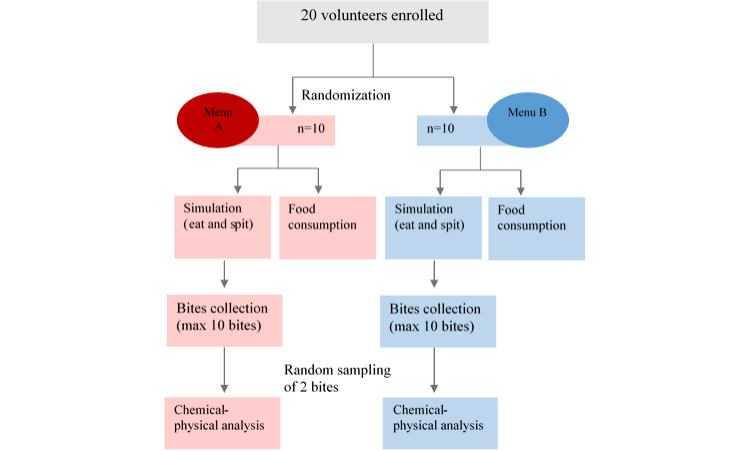
NOTION study design and flowchart.

A selection of different types of prepackaged foods will be made by dieticians to simulate a complete Mediterranean daily diet of 2000 kcal or less. The choice of prepackaged food is driven by the fact that bias regarding the energy content will be reduced because the caloric information reported on the labels will be compared to the values measured by the calorimeter. Two menus (menus A and B, Table 1) of Italian food items will be set up and randomly assigned to the volunteers. Volunteers will be asked to be present on two different days at fixed times to have breakfast and lunch on the first day, and snack and dinner on the second day. Each volunteer will wear two devices, one on each wrist, and eat the assigned food in two stages: first simulated and then the actual meal right after. During the simulated meal, the participants have to simulate a maximum of 10 bites for each food item without ingesting it. The operators will collect the bites in plastic bags, previously labeled with a unique alphanumeric code designed to allow the traceability of each bite along all the experimental phases from the collection to the final storage. During the actual meal, participants will be asked to eat the meal continuously and naturally. All stages will be videotaped. The several stages are shown in Figure 1.

### Tools

Two digital cameras will be used to record each participant’s mouth, torso, and tray during meal consumption. Four Garmin Fenix 5 watches containing an accelerometer and gyroscope will be used to record the movement of the wrists of each participant at 5 Hz. The FIT files generated by the watches will be converted using the java/FitToCSV.bat utility in the FIT SDK. They will contain all the relevant information for the analysis: triaxial accelerometer data x, y, z, pitch and roll angle, power, and time.

Gold standard evaluation of the energy content of selected foods will be obtained by calorimetric assessments made at the Laboratory of Dietetics and Nutraceutical Research of the Department of Cardiac, Thoracic and Vascular Sciences of the University of Padova, Padova, Italy.

For this purpose, all collected bites will be measured by mass, while two bites of each food item per participant will be randomly selected to measure volume and energy content. The mass of the bites will be measured using the Gibertini Crystal 100 scale. The volume measurement will be obtained measuring the liquid displacement due to the immersion of the sample in a graded cylinder (250 mL class A) filled with water. Caloric content of bites will be measured using a bomb calorimeter after the complete homogenization of a single bite [18]. Each sample will be analyzed in duplicate. Every 30 burns the benzoic acid standard will run to ensure the instrument calibration [19,20]. 

**Table 1:** Composition of the two menus (menus A and B) randomly assigned to the volunteers recruited in the NOTION study.

Menu and meal			kcal/portion
**Menu A**			
	**Breakfast**		
		Rusks and jam	114 kcal/41 g
		Yogurt	186 kcal/170 g
	**Lunch**		
		Risotto with asparagus	471 kcal/300 g
		Mozzarella	242 kcal/100 g
	**Snack**		
		Biscuits	278 kcal/55 g
	**Dinner**		
		Vegetable soup	135 kcal/310 g
		Artichoke chicken	199 kcal/120 g
**Menu B**			
	**Breakfast**		
		Brioche	99 kcal/28 g
		Yogurt	186 kcal/170 g
	**Lunch**		
		Tagliolini with mushrooms	546 kcal/300 g
		Chicken meatballs with tomato sauce	135 kcal/120 g
	**Snack**		
		Sandwich	195 kcal/70 g
	**Dinner**		
		Eggplant parmigiana	309 kcal/300 g
		Italian fresh cheese	269 kcal/100 g

The calorimeter calculates the gross energy of the samples; therefore, to consider the metabolizable energy it will be necessary to correct the value by subtracting 1.25 kcal per gram of protein from gross energy.

### Randomization and Sampling

Each volunteer will be assigned a menu through randomization, providing two balanced groups of 10 participants for each menu. A sampling strategy will be applied to select 2 of 10 bites collected during the simulated meal for chemical and physical analysis.

### Randomization to Food Menus

With the aim to randomize each participant to a menu, the “blockrand” package of R will be used. This function creates random assignments for clinical trials, or any experiment, in which subjects are enrolled sequentially. Blocked randomization with a 1:1 ratio will be performed to ensure balance between the groups throughout the study.

### Sampling of the Bites

Bites per food item and participant will be numbered progressively from 1 to 10 as they are collected. After random reordering, performed with the “sample” function of R, the first two will be sampled. Since sampling is scheduled in advance, and a participant could complete the meal in less than 10 bites, if the bite indicated in the reordered list be unavailable, then the next in the list will be considered (eg, assume that the list is 1,10, 3, 2, 7, 4, 8, 5, 9, 6 and the participant has finished the meal in five bites, then the first and third bites will be analyzed).

### Data Processing

#### Bite Identification

A human rater will annotate an Excel file by watching the video and pausing it at times when a bite is seen to be taken, using frame-by-frame rewinding to identify the time when food or a beverage is placed into the mouth. Two human raters independently will label each food. Raters will be trained to standardize the process of labeling.

#### Sensor and Video Synchronization

To achieve synchronization without wires of cameras and wrist motion trackers, device clocks will be synchronized with the participants’ watch times at the beginning of each data collection session. Participants will be asked to clap their hands at the beginning of each eating session. Watch times will be manually recorded for the periods of clapping. The peaks of the distinct sinusoidal patterns at the beginning of each acceleration signal will be visualized on the video and aligned between the wearable devices.

#### Signal Preprocessing

Working with sensor signals coming from wearable devices that monitor human movements involves the use of signal processing techniques to remove noise from the data [21]. As a result, the accuracy of a predictive model built on preprocessed data will depend on the choice of the most suitable filtering parameters. Another important aspect that should be considered is the computational cost of performing both sensor preprocessing and model fitting. There is great interplay between signal preprocessing and model performance. Different methods to preprocess sensor data will be evaluated and compared in terms of effectiveness to extract the maximum information at a low computational cost [21].

Row acceleration signals consist of movement, gravitational, and noise components. Separation of these becomes difficult during rotational movements [22]. These two components overlap in the frequency domain so cannot be completely separated by filtering. However, most of the acceleration of human body movements occurs below 0.2 to 0.5 Hz, and thus a reasonable estimate of separation will be carried out using a Butterworth low-pass filter with 0.3 Hz cutoff frequency [16,22,23]. Usually after applying the noise filter, feature extraction occurs for classification [23,24]. A vector of features will be obtained by calculating variables from the time and frequency domain on sliding windows with a percentage of overlap [25,26]. As an alternative to sensor preprocessing, we will apply the discrete-time Fourier transformation to each sensor data [27]. This choice is because data needs to be transformed into frequencies, so they are no longer dependent on time. Therefore, we will be able to decompose the time series into single values that correspond to every single variable in the classification model.

### Algorithm

#### Machine Learning Techniques

The use of accelerometer data has recently emerged in several biomedical apps (eg, [28,29]). Researchers have developed apps, such as those for fitness wearables, with low-level software design to perform both signal processing and analysis operations. Supervised machine learning techniques have proven to be very useful in this context, with an excellent ability to recognize human movements [30,31]. Starting from these results, we will test several machine learning techniques to recognize the food items, and then estimate the caloric intake conditional on the identification of the food. We will formulate food recognition as a classification problem, where the classes represent the food items. We will evaluate different classifiers, among them random forest, bagging, weighted k-nearest neighbor, and support vector machine. Supervised learning training, with cross-validation techniques to access the best model in terms of accuracy, will be performed. Finally, an assessment of the model prediction will be made for test data.

#### Estimation of Caloric Intake

The purpose of this study is obtaining an estimate of caloric intake from the movements of the wrist. To achieve this objective, we will exploit accelerometer data because it is known to be a suitable adoption for this kind of problem [29,32]. Compared to past work, we will not limit ourselves to counting the number of bites [11], but we will try to identify the type of food to better predict its caloric value. We will explore several algorithms trained in a supervised way to obtain a recognition of food items from accelerometer data. Then, we will estimate a caloric intake starting from an identified bite with a supervised learning technique. We will use a two-step algorithm: first, we estimate which kind of food people eat, and then we predict a mean caloric intake for each bite identified conditional on the food item.

## Results

Building an automatic calculation method for caloric intake independent of the black-box processing of the wearable devices marketed so far, has great potential both for clinical nutrition (eg, for assessing cardiovascular compliance or for the prevention of coronary heart disease through proper dietary control) and public health nutrition as a low-cost monitoring tool for eating habits of specific segments of the population.

From this study, we expect to obtain a series of relevant data that, opportunely filtered, could allow the construction of a prototype algorithm able to estimate caloric intake through the recognition of food type and the number of bites. The algorithm should work in real time and be embedded in a wearable device able to match bite-related movements and the corresponding caloric intake with high accuracy. The challenge will be refining the algorithm to recognize an extended number of foods on a large sample of multiethnic participants.

## Discussion

### Overview

The estimation of caloric intake is a topic of great interest in the field of nutrition [1,2]. Monitoring the consumption levels of food can be important for those who care about their body, but it becomes crucial when the impact of diet on the progression of some diseases, such as obesity, diabetes, and cardiovascular diseases, is the focus [33]. Several tools are currently available, but their effectiveness is limited to a gross measurement of the caloric intake, which is strongly influenced by the accuracy and the memory of the interviewee [1,2,7]. The main applications of automatic monitoring systems on nutrition intake are to avoid subjective influences in manual reports and to provide a comfortable and accurate way for controlling food intake in daily life. Although the instruments based on data recorded by motion sensors and wrist accelerometers overcome the limits related to the lack of objectivity in the quantification of foods and portions, they cannot completely fill the gap between the recognition of the bites and their respective energy contribution [8,13-16].

### Strengths

To handle the challenges of big data from wearable devices, new statistical thinking and computational methods are needed. The use of machine learning techniques, able to grab all the available information to solve complex learning tasks such as classification, clustering, and numerical prediction, represent a possible advancement.

This study will address these existing challenges and make the following innovative contributions:

1. Build a detailed and objective picture of eating activity under seminaturalistic conditions, as a reliable and robust basis for the subsequent analytical steps.
2. Use cutting-edge advances in machine learning for big raw data generated by inertial sensors.
3. Implement a prototype algorithm for caloric intake, independent on the wearable device used at the development stage, which will hopefully improve dietary monitoring.

### Limitations and Final Remarks

We acknowledge that the whole system is still premature for real-time implementation and that future work will require a broader range of food items, a larger sample size, and a free-living condition. Furthermore, we will need to check the hypothesis that the type of food rather than other participant and food characteristics (eg, cutlery used to eat, anthropometry) is the best proxy of caloric intake. Moreover, we will have to investigate whether the signals recorded by the wearable devices actually hold promise in measuring caloric intake. Nevertheless, this ambitious project has great potential to empower dietary monitoring.
